# Anti-microbial activity of whole blood and plasma collected from Anna’s Hummingbirds (*Calypte anna*) against three different microbes

**DOI:** 10.1371/journal.pone.0234239

**Published:** 2020-06-11

**Authors:** Andrea M. DeRogatis, Leilani V. Nguyen, Ruta R. Bandivadekar, Kirk C. Klasing, Lisa A. Tell

**Affiliations:** 1 Department of Animal Science, University of California, Davis, California, United States of America; 2 Department of Medicine and Epidemiology, School of Veterinary Medicine, University of California Davis, Davis, California, United States of America; USDA-Agricultural Research Service, UNITED STATES

## Abstract

Hummingbirds are essential pollinators in many ecosystems, making their conservation critical. As is the case with many species, hummingbirds are now facing a variety of challenges resulting from anthropogenic changes. As populations shift and species interactions change, disease is likely to pose a significant threat. There is a basic understanding of which pathogens currently affect a variety of hummingbird species, however there is a paucity of information about their immune systems capacity to kill pathogens and what specific factors may affect immunity. The objective of this study was to gain a basic understanding of the effect of age, sex, and molt on the constitutive innate immunity of hummingbirds. An *in vitro* assay was used to assess the microbiocidal capacity of the whole blood of Anna’s Hummingbirds (*Calypte anna*) against three different microbes: *Escherichia coli* (*E*. *coli*), *Staphylococcus aureus* (*S*. *aureus*) and *Candida albicans* (*C*. *albicans)*. The effect of age, sex and molt on anti-microbial capacity varied based on the microbe type. After-hatch-year birds tended to have better anti-microbial capacity compared to hatch-year birds. Male birds had higher anti-microbial activity than female birds, although this was not observed against *C*. *albicans*. Molting birds had a weaker antimicrobial activity against *E*. *coli* and *S*. *aureus* than birds that were not molting. These results represent an important first step towards defining the parameters of constitutive innate immunity of Anna’s Hummingbirds as well as providing important knowledge about factors that should be considered when evaluating the health of wild populations.

## Introduction

Hummingbirds are very unique organisms. They are known for their high metabolic rates and have adapted to thrive at physiological extremes that many animals are unable to tolerate [1, 2, 3]. Besides bats, they are one of the only vertebrate pollinators, making them essential for a variety of ecosystems across the globe [4, 5]. As different anthropomorphic affects begin to threaten hummingbirds, conservation efforts are becoming increasingly important. In order for conservation efforts to be successful, it is necessary to establish what the normal health parameters are for a variety of hummingbird species. Defining normal health parameters for hummingbirds involves not only understanding the disease susceptibility of populations and species, but also the underlying function and capabilities of the hummingbird immune system. By studying free-ranging hummingbird populations, it is possible to get a more complete understanding of the effects of factors such as age, migration status or molt status on the immune system [6].

There has been minimal research done to understand what factors may influence hummingbird health and to characterize the specific disease threats that hummingbirds face. Through surveillance activities, it has been reported that hummingbirds test positive for West Nile virus and are susceptible to avian pox [7, 8]. To date, there is very little data on bacterial pathogens affecting hummingbirds with only four species recorded as pathogenic to hummingbirds including *Chlamydia psittaci*, *Mycobacterium avium*, *Salmonella typhimurium*, and *Serratia marcescens* [9, 10]. Four species of fungi have also been recorded as pathogens of hummingbirds, including a *Candida* species [11]. Comparatively, there are an abundantly greater number of eukaryotic parasites identified in hummingbirds. There are currently 23 species of parasites including helminths, protozoa and ectoparasites that are known to parasitize hummingbirds [11, 12]. Many of the pathogens of hummingbirds have only been reported once in the literature, demonstrating the necessity of expanding the knowledge of diseases likely to threaten hummingbird populations.

In order to fully evaluate the risk an animal faces from disease, basic knowledge of an animal’s immune system is essential. The only immunological work done in hummingbirds has shown that both hatch-year Anna’s Hummingbirds (*Calypte anna)* and Black-chinned hummingbirds (*Archilochus alexandri*) have higher lymphocyte counts compared to adult birds [13]. In the ecology and immunology literature, there is a developing theory that both organism body size and metabolic rate correlate with immune function [14, 15]. It is nutritionally costly to mount an immune response; therefore, it is thought that animals with higher metabolisms are more likely to invest in constitutive immunity to reduce the cost associated with ramping up an adaptive immune response [16]. Investigating the immune system of hummingbirds could provide further insight into the interaction between metabolism and investment in different components of the immune system.

Overall, there is a compelling need for more information about not only the diseases of hummingbirds but also how their immune systems function. Fortunately, there are immunological assays that have been developed to study free-ranging avian species that could be applied to hummingbirds to provide insight into the capacity of their immune systems. Some of these immunological assays can be adapted for use with less than 10 microliters of whole blood and could be safely used with hummingbirds even when taking into consideration their size as a limiting factor [17, 18]. One such assay, the anti-microbial assay, uses whole blood to study the anti-microbial activity of both phagocytic cells and protective proteins found in the plasma. When performed with different microbes, this assay can provide useful insight into the capacity of an organism’s constitutive innate immune system [18]. The objective of this study was to use the anti-microbial assay to evaluate the constitutive innate immunity of Anna’s Hummingbirds and determine which factors may be most important to consider when performing future health and disease studies. Here we documented how different factors, such as sex or molt status, influenced the anti-microbial activity of both the whole blood and plasma of Anna’s Hummingbirds.

## Materials and methods

### Blood collection

Anna’s Hummingbirds (*Calypte Anna)* were captured in three geographically similar locations in Yolo county located in Northern California (Winters; 38° 31′ 49″ N, 121° 51′ 2″ W; 38° 28' 53.2" N, 122° 02' 0.8" W; 38° 29' 2.5" N, 121° 59' 45" W). All three study sites were located on private property and permission to access study sites was granted by the homeowners. Due to the study sites being located on private property, no permits were required for land access and GPS coordinates are approximated to the nearest major intersection. Anna’s Hummingbirds were captured using drop net feeder traps during May, June, August and September of 2018.

Sterile blood samples were collected via a single toenail clip. Blood samples were collected within five minutes of capture to reduce the potential effect of stress on the immune system. To generate a sterile bleed site, prior to blood collection, the hummingbird’s foot was cold sterilized with 70% ethanol. Once the 70% ethanol had dried, sterilized curved surgical scissors were used to remove less than 1 mm from the tip of the toenail. Blood was then collected directly from the clipped toenail into a sterile heparinized microhematocrit capillary tube (Fisherbrand #22362566). Following blood collection, the microhematocrit tube was rocked back and forth to expose the blood to the heparin to prevent clotting. The blood was then centered in the microhematocrit tube and the microhematocrit tube was sealed using a clay card (Fishersci #0267620) that had been sterilized with 70% ethanol. The blood sample collection volumes ranged from 15 to 40 μL of whole blood. In September 2018, all blood samples collected were used to sample plasma. For plasma samples, whole blood was collected using the same above described sterile method. Sealed microhematocrit tubes were centrifuged for five minutes at 11,500 rpm using a micro-capillary centrifuge (IEC International SKU # ST0036257). Once a blood sample had been collected, the hummingbird was examined, and the species, sex and age of each bird was determined using guidelines from the North American Hummingbird Manual [19]. Birds were categorized to be molting if any of the remiges or rectrices were in the process of being replaced bilaterally. All animal use procedures were approved by the United States Fish and Wildlife (Permit: MB55944B-2), United States Geological Survey Bird Banding Laboratory (Permit: 23947), California Department of Fish and Wildlife (Permit: SC-013066) and the UC Davis Institutional Animal Care and Use Committee (Protocol: 20355).

### Anti-microbial assay

The anti-microbial assay protocol is based off the protocol used by Millet et al. (2007) for small blood volumes [18]. Three different lyophilized microorganism pellets were used for the anti-microbial assay: *Escherichia coli* (American Type Culture Collection (ATCC)^®^ #51813^™^; Microbiologics^™^ #0791L), *Staphylococcus aureus* (ATCC^®^ #6538^™^; Microbiologics^™^ #0458L) and *Candida albicans* (ATCC^®^ #10231^™^; Microbiologics^™^ #0443L). These microbes were isolated from mammals and are not known to be avian pathogens. The microbes were selected to serve as a Gram-negative, Gram-positive or fungal challenge of the innate immune system. They are commonly used in comparative studies of vertebrate immunity [18, 20, 21]. The aim was to test each blood sample against each of the three microbes, however if a low volume of blood was collected only a single microbe was used for a given sample. Per the manufacturer’s instructions, lyophilized pellets were reconstituted in 40 mL sterile endotoxin-free phosphate buffered saline (PBS) for a final concentration of 2.5 x 10^5^ CFU/mL. The reconstituted microbial stock solutions were stored at 4°C for up to seven days. Prior to use each day, a working solution of *E*. *coli* and *S*. *aureus* (2.5 x 10^4^ CFU/mL) was prepared by diluting the microbial stock solution ten-fold with sterile endotoxin free PBS. The undiluted stock solution of *C*. *albicans* was used for all *C*. *albicans* experiments.

Once the whole blood was collected in the field, the whole blood was immediately diluted at a 1:4 ratio with CO_2_-independent media (Thermo Fisher #18045088) plus 4 mM L-glutamine (Gibco #25030081). The CO_2_-independent media was pre-warmed in a water bath to 41°C. The whole blood, CO_2_-independent media and microbial working solution were then combined in sterile 0.5 mL microcentrifuge tube and vortexed. The diluted blood microbe mixtures containing either *E*. *coli* or *S*. *aureus* were then incubated in a 41°C water bath for 30 minutes. The diluted blood combined with *C*. *albicans* was incubated for two hours at 41°C. After the incubation time was completed, samples were removed from the water bath and placed directly onto ice for five minutes to stop any additional microbe-blood interactions. The entire sample volume was then plated onto a tryptic soy agar (TSA) plate and incubated for 24 hours at 37°C for *E*. *coli* and *S*. *aureus* samples and 48 hours at 37°C for *C*. *albicans*. Throughout each sampling day, control plates were prepared by plating 5, 10 or 15 μL of the microbial working solution onto TSA plates. Additional controls were prepared by diluting the microbial working solution in either CO_2_-independent media or tryptic soy broth (TSB) and incubating the sample at 41°C for 30 minutes for *E*. *coli* and *S*. *aureus* or for two hours for *C*. *albicans*. All controls were plated onto TSA plates and incubated using the protocol described above for the experimental sample plates. The antimicrobial results are reported as a percent change in the CFU at the end of the incubation relative to the initial CFU inoculated ((viable number of microbes post incubation—initial microbe inoculation)/initial microbe inoculation). Thus, a negative value indicates microbial killing and a positive value indicates growth of the microbe.

### Statistics

All statistical analyses were performed using Microsoft Excel^®^ 2016, R© version 3.5.1 and RStudio © version 1.1.463. General linear models (GLMs) were used to determine the effect of age, sex, molt on the anti-microbial capacity of the blood. GLMs were generated for each microbe separately due to the differences in the scale of the anti-microbial activity relative to each microbe. The final model for both *E*. *coli* and *C*. *albicans* included the main effects of age, sex and molt. The GLM for *S*. *aureus* included terms for age, sex, molt as well as an interaction term for age and molt status.

For each microbial data set, the normality of the data distribution and equality of variance were checked using the Shapiro-Wilk Test and through examination of the model residuals. Bonferroni significance levels were used to determine outliers based on the linear models used for each microbial data set. A single data point was considered an outlier and was therefore removed from the whole blood *E*. *coli* data set and two outliers were removed from the whole blood *S*. *aureus* data set. The statistical analysis was repeated with and without the outliers, with no changes in statistical interpretation observed. The data from a single study date were not included due to assay parameters being outside of normal limits. For each main factor or factor interaction, the least-squared mean was calculated. Analysis of Variance (ANOVA) was used to evaluate the main effect of age, sex and molt as well as the interaction of age, sex and molt where appropriate (*P* ≤ 0.05). When an interaction term was kept in a model, the significances between the interacting means were calculated using a Tukey procedure *P*-value correction.

## Results

### Sample demographics

A total of 116 blood samples were collected from Anna’s Hummingbirds over the course of the study (Table 1). Depending on the volume of blood collected from each individual, the anti-microbial activity of the blood was tested against *E*. *coli* (n = 116), *S*. *aureus* (n = 86) and/or *C*. *albicans* (n = 18).

**Table 1:** Demographic composition of Anna’s Hummingbirds used to evaluate the anti-microbial activity of blood against three different microbial species. The number of blood samples tested from Anna’s Hummingbirds for each classification of sex, age, molt status and blood type are shown.

	Male	Female	After-hatch-year	Hatch-year	Molt	No Molt	Plasma	Whole Blood
*E*. *coli*	48	43	48	44	56	36	12	12
*S*. *aureus*	31	31	28	35	56	7	12	12
*C*. *albicans*	10	8	3	15	16	2	0	0

### Anti-microbial activity of the whole blood

#### *E*. *coli*

**Fig 1:**
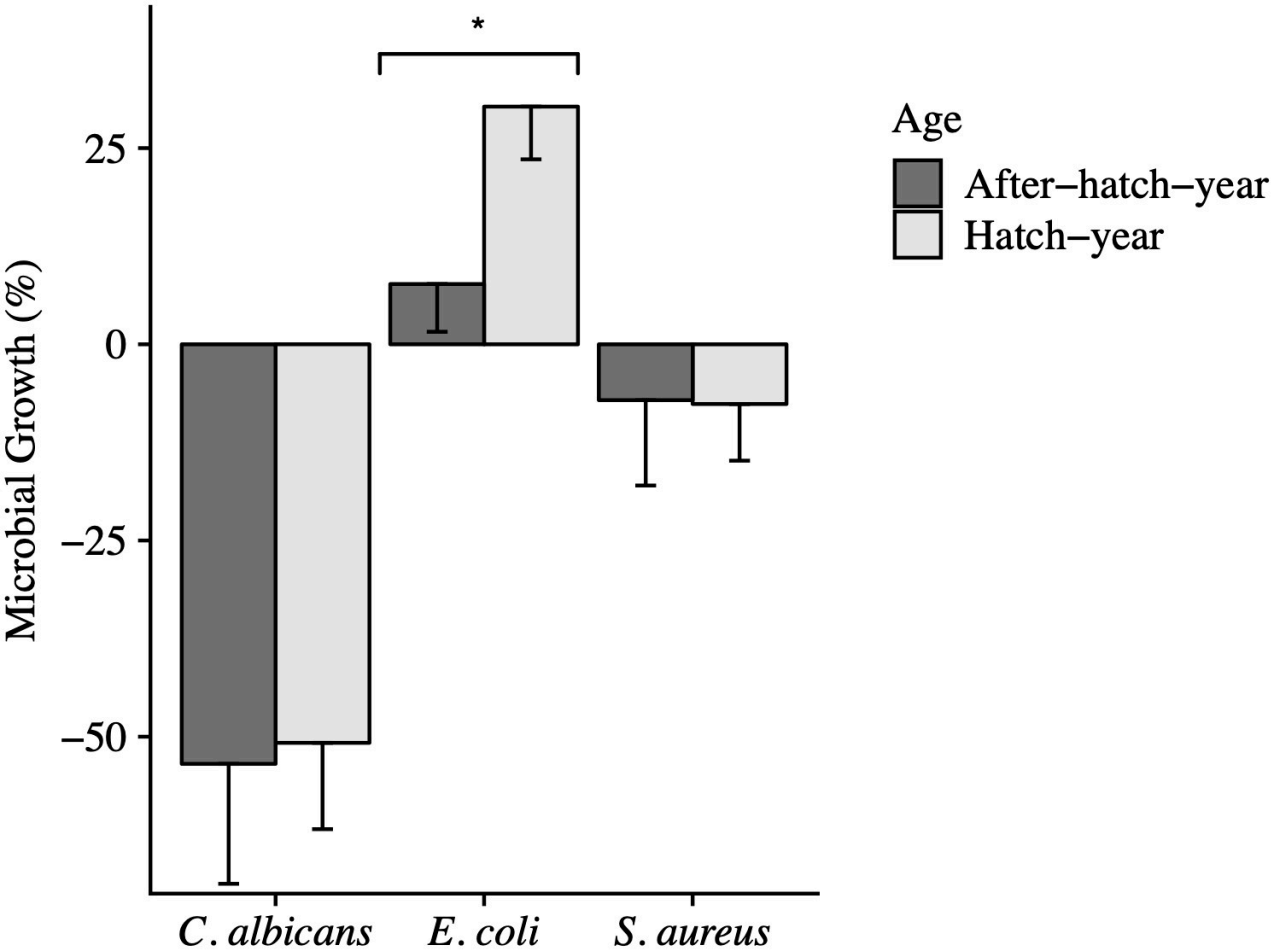
The effect of age on the anti-microbial activity of whole blood from Anna’s Hummingbirds. Error bars represent the standard error of the mean (SEM). There was no difference in the killing ability of whole blood from after-hatch-year birds (n = 3) compared to hatch-year birds (n = 15) against *C*. *albicans* (*P* = 0.83). Age also did not affect the activity of whole blood from after-hatch-year hummingbirds (n = 28) compared to hatch-year birds (n = 35; *P* = 0.46). After-hatch-year birds (n = 48) killed significantly more *E*. *coli* than hatch-year birds (n = 44; *P* = 0.007).

**Fig 2:**
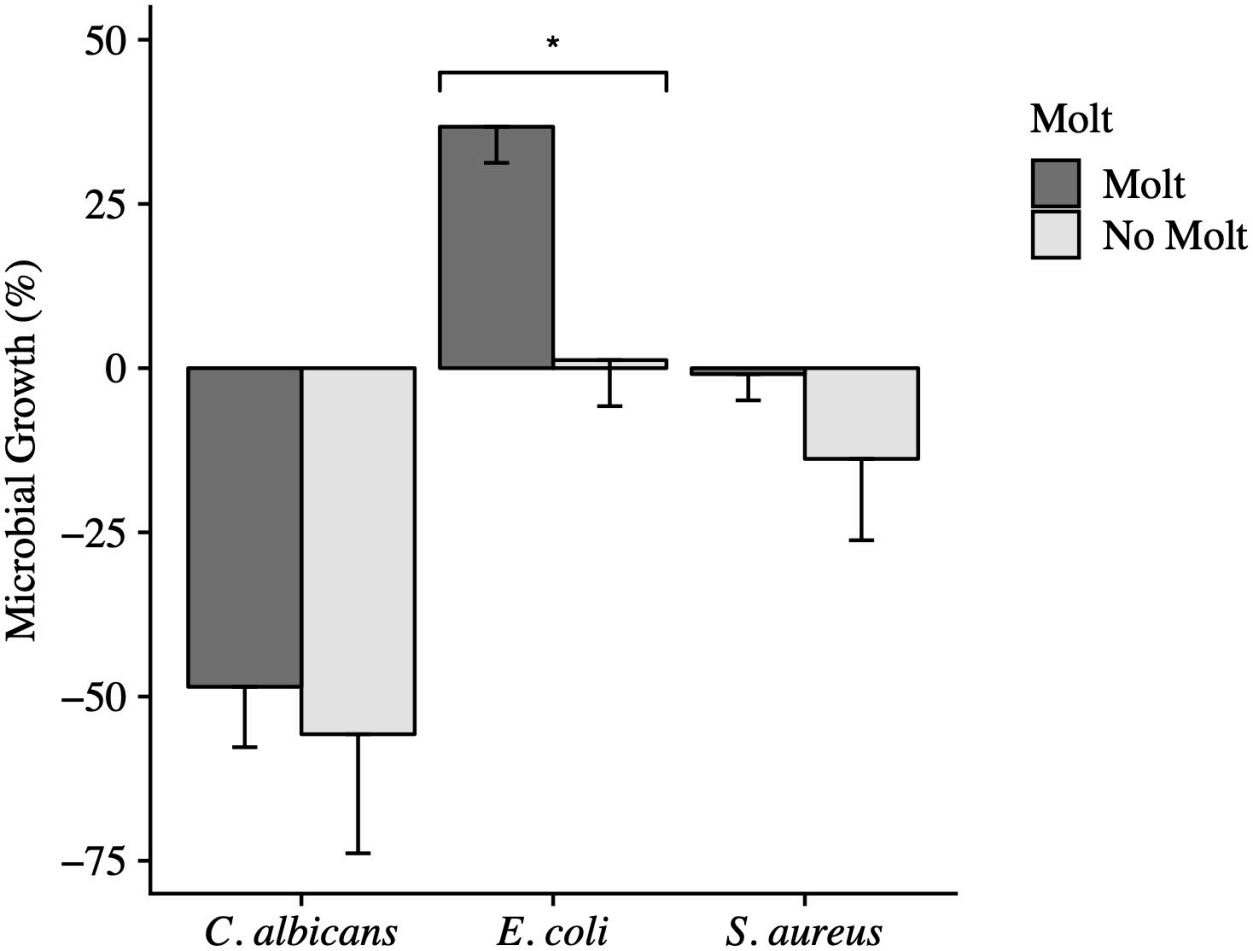
The effect of molt status on the anti-microbial activity of whole blood from Anna’s Hummingbirds. Error bars represent the standard error of the mean (SEM). Molting birds (n = 16) did not differ in their ability to *C*. *albicans* from hummingbirds not molting (n = 2; *P* = 0.73). Molting birds (n = 56) had significantly lower anti-microbial activity against *E*. *coli* than birds not molting (n = 36; *P* < 0.001). Birds that were not molting (n = 7) tended towards killing more *S*. *aureus* than molting birds (n = 56), but the difference was not significant (*P* = 0.09).

**Fig 3:**
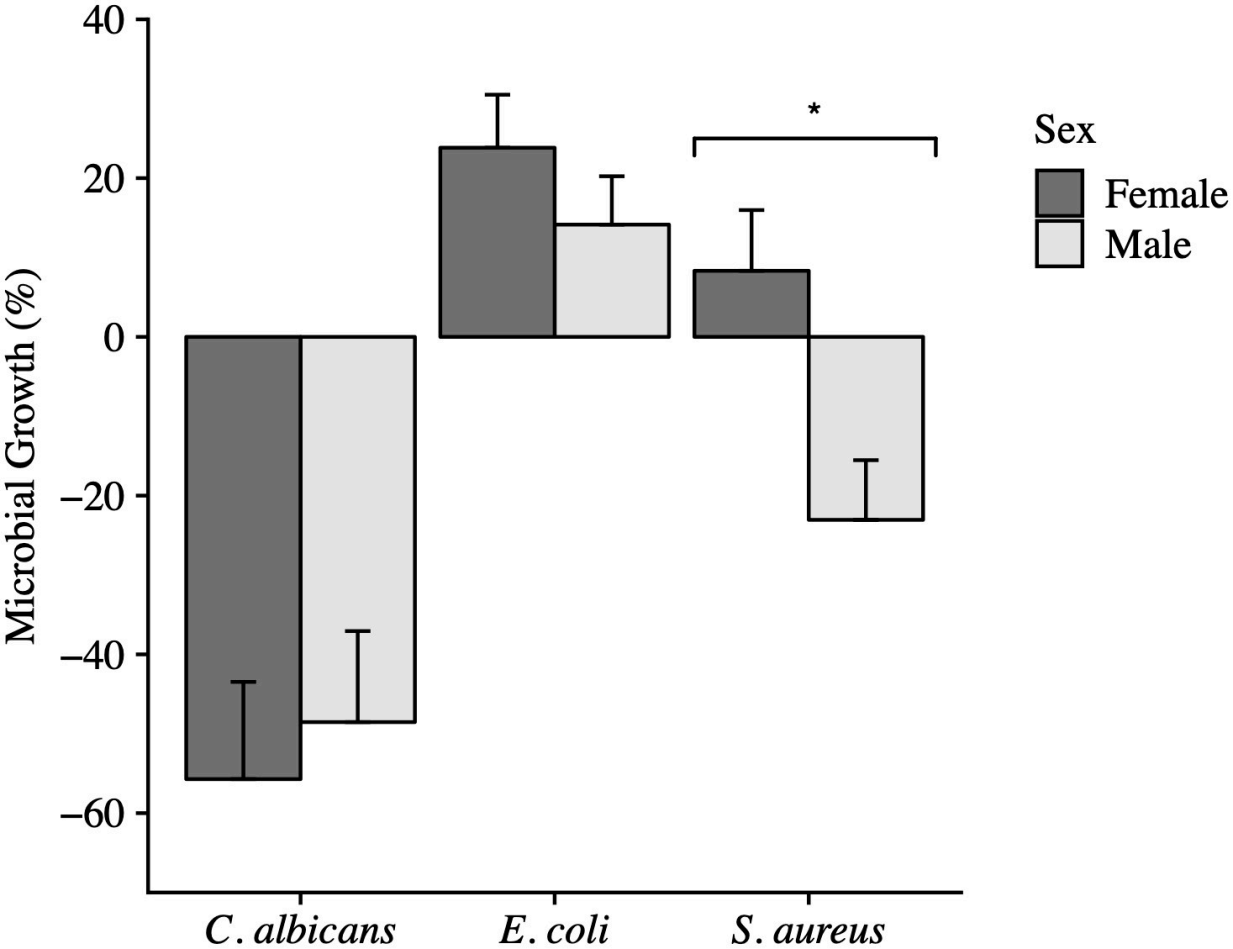
The effect of sex on the anti-microbial activity of whole blood from Anna’s Hummingbirds. Error bars represent the standard error of the mean (SEM). There was no significant difference in the anti-microbial activity of the whole blood of male (n = 10) and female (n = 8) birds against *C*. *albicans* (*P* = 0.55). Male birds (n = 48) trended towards killing more *E*. *coli* than female birds (n = 43; *P* = 0.12). Male birds (n = 31) killed significantly more *S*. *aureus* than female birds (n = 31; *P* < 0.001).

The anti-microbial activity of the whole blood against *E*. *coli* was significantly affected by a bird’s age (*P* = 0.007). Anna’s Hummingbirds classified as hatch-year birds inhibited *E*. *coli* growth, on average, 22.6% less than birds classified as after-hatch-year (Table 2; Fig 1). Molt status also significantly influenced the anti-microbial activity of whole blood against *E*. *coli* (*P* = 0.0001; Fig 2). Hummingbirds that were molting inhibited *E*. *coli* growth 35.5% less than non-molting birds. The sex of a hummingbird trended towards but did not significantly influence the hummingbird’s ability to kill *E*. *coli* (*P* = 0.12; Fig 3). Female hummingbirds inhibited *E*. *coli* growth 9.7% less than male hummingbirds (Table 2).

**Table 2:** Growth of three different microbial species following exposure to whole blood collected from Anna’s Hummingbirds. The average microbial growth and standard error of the mean (SEM) of three different microbes when exposed to the whole blood of Anna’s Hummingbirds varies based on sex, age, molt status and the microbe challenge. Values shown represent a percent change in microbial growth, where a negative value indicates a decrease in the percentage of microbial growth following incubation with whole blood.

	*E*. *coli*	*S*. *aureus*	*C*. *albicans*
Male	14.1 ± 6.1	-23.1 ± 7.7	-48.5 ± 11.5
Female	23.8 ± 6.8	8.3 ± 7.5	-55.7 ± 12.2
After-hatch-year	7.7 ± 6.1	-7.1 ± 10.9	-53.5 ± 15.3
Hatch-year	30.3 ± 6.7	-7.6 ± 7.2	-50.8 ± 11.0
Molt	36.8 ± 5.5	-0.9 ± 4.0	-48.5 ± 9.2
No Molt	1.2 ± 7.0	-13.8 ± 12.4	-55.7 ± 18.1

#### *S*. *aureus*

Neither the age (*P* = 0.46) nor the molt status of a hummingbird alone had a significant effect on anti-microbial activity. Of the two factors, molt status had the strongest effect compared to age and trended towards significance (*P* = 0.09; Table 2; Figs 1 and 2). The sex of the hummingbird had a strong effect on the ability of the bird to kill *S*. *aureus* (*P* = 0.001; Fig 3). Male hummingbirds killed, on average, 31.4% more bacteria than female hummingbirds (Table 2, Fig 3). The interaction of age with molt status neared significance (*P* = 0.14; Table 3). This is because during molt, after-hatch-year birds killed 19.1% more *S*. *aureus* than hatch-year birds (*P* = 0.0497). When after-hatch-year or hatch-year birds were not molting, there was no difference in anti-microbial activity due to age (*P* = 0.84).

**Table 3:** Main effects of hummingbird age and molt status on the anti-microbial activity of whole blood against *S*. *aureus*. The average microbial growth, as a percent change, of *S*. *aureus* following exposure to the whole blood of Anna’s Hummingbirds is shown. A negative value indicates a decrease in microbial growth. The P-values associated with the main effects of age, sex, molt and the interaction of age with molt are shown.

Age	Molt Status	Microbial Growth (%)	SEM
After-hatch-year	Molt	-10.444743*	5.927302
After-hatch-year	No Molt	-3.785681	20.92904
Hatch-year	Molt	8.625632*	5.576199
Hatch-year	No Molt	-23.849469	13.259628
***P*-Value**			
Age		0.46	
Sex		0.001	
Molt		0.09	
Age*Molt		0.14	

*Numbers followed by an asterisk differ significantly (*P* < 0.05).

#### *C*. *albicans*

Neither age, molt nor sex had a significant effect on the anti-microbial activity of whole blood against *C*. *albicans* (Figs 1, 2 and 3). On average, after-hatch-year birds killed 2.7% more *C*. *albicans* than hatch-year birds (*P* = 0.83; Table 2). Female birds killed 7.2% more yeast than male birds (*P* = 0.55; Table 2). When comparing the molt status of individuals, non-molting birds killed 7.2% more *C*. *albicans* than molting birds (*P* = 0.73; Table 2).

#### Plasma vs. whole blood

**Fig 4:**
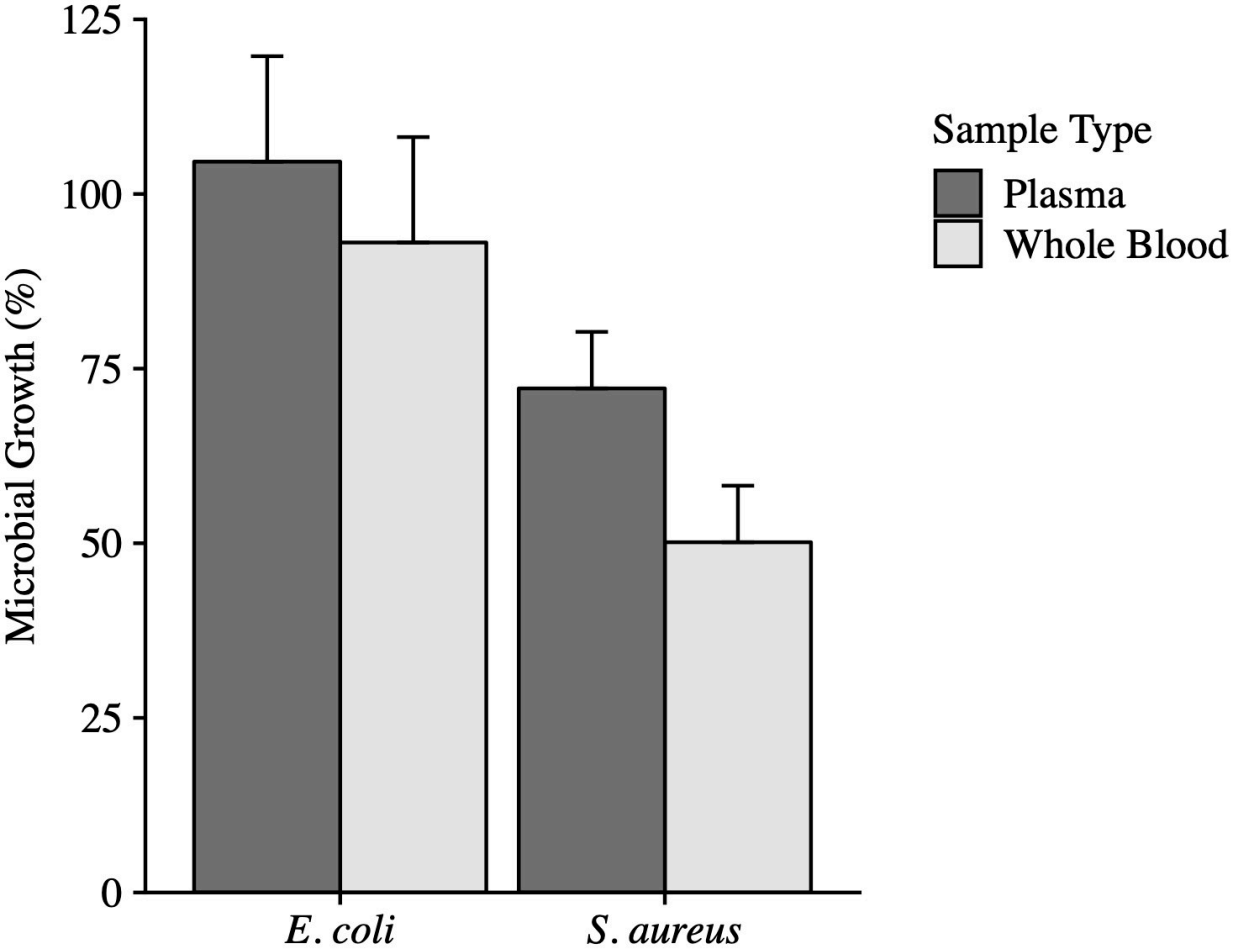
Anti-microbial activity of whole blood compared to plasma collected from Anna’s Hummingbirds. Error bars represent the standard error of the mean (SEM). The anti-microbial activity of plasma (n = 12) or whole blood (n = 12) did not differ against *E*. *coli*. Whole blood (n = 12) trended towards having higher anti-microbial activity against *S*. *aureus* than plasma (n = 12; *P* = 0.07).

Whole blood inhibited *E*. *coli* growth on average 11.6% more than the plasma but this difference was not significant (*P* = 0.59). Whole blood tended (*P* = 0.07) to kill 22.0% more *S*. *aureus* than plasma alone (Fig 4).

## Discussion

The objective of this study was to begin to characterize the constitutive innate immunity of Anna’s Hummingbirds and the effect of age, sex, and molt on constitutive innate immunity. In general, age, sex and molt status affected anti-microbial activity of the whole blood against *E*. *coli* more than *S*. *aureus* or *C*. *albicans*. Anna’s hummingbird had the greatest anti-microbial activity against *C*. *albicans* compared to *E*. *coli* and *S*. *aureus*. Anna’s hummingbirds appear to have stronger constitutive innate immunity against fungi compared to gram-negative or gram-positive bacteria. *Candida* sp. are a known pathogen of hummingbirds [9]. As pollinators, hummingbirds are continually exposed to the microbiomes of the flowers they collect nectar from [22]. The fungal taxa of nectar are unique compared to the fungal taxa of both hummingbirds and hummingbird feeders [23]. As pollinators, hummingbirds may have a higher exposure to fungal species compared to other avian species and have therefore developed stronger constitutive immunity against fungus.

After-hatch-year birds generally had higher anti-microbial activity against all three microbes compared to hatch-year birds, however the trend was only significant for *E*. *coli* (*P* = 0.007). The development of immunity in Anna’s Hummingbirds has not been studied, but the observed difference could be due to immaturity of the immune system in the hatch-year birds. Alternately, the difference could be due to a higher level of competition for resources between immunity and maturation in the younger hatch-year birds. When considering the effect of sex, male birds tended to have greater microbial killing of *E*. *coli* and *S*. *aureus* (*P* = 0.001), but not *C*. *albicans*. During the sampling period of the study, May–September, female birds were likely to be rearing or have just finished rearing young birds. The tradeoff between the very high costs of reproduction with immunity for female hummingbirds could account for the difference in anti-microbial activity observed when comparing the sexes [24].

When considering the molt status of a bird, the general trend was that birds not molting had greater anti-microbial capacity compared to molting birds. When birds are molting, the nutritional cost of growing feathers is likely to compete with the costs of mounting an immune response [25]. Birds that were not molting may have more nutritional resources available to allocate towards the immune system [26]. When the hummingbirds were molting, older after-hatch-year birds had greater anti-microbial activity than younger hatch-year birds. However, there was no difference in anti-microbial activity between hummingbirds of different ages when the birds were not molting. During our summer study period, hatch-year hummingbirds were still undergoing growth and development. Age and molt status tended to interact because both of growth and molt are both energetically costly processes. The decrease in anti-microbial activity when hatch-year birds are molting indicates the presence of a trade-off between the energetic demands of molt, growth and immunity. After-hatch-year birds were not subject to the same costs associated with growth and could likely maintain higher levels of constitutive innate immunity during molt, indicated by their greater anti-microbial activity. When hatch-year birds were not under the constraints of molt, the pressure of a trade-off across processes was reduced and both age groups had similar anti-microbial activity during the period when neither were molting. We were able to obtain fewer samples from non-molting birds when working with *S*. *aureus*, which made it difficult to detect a clear trend for the non-molting birds. Due to the smaller sample size, the standard error for the non-molting hatch-year birds is very large. In the future, it would be important to obtain additional samples from non-molting birds to clarify the anti-microbial activity of birds not molting against gram-positive bacteria such as *S*. *aureus*.

As part of our study, the anti-microbial activity of the whole blood was compared to plasma alone. When whole blood is centrifuged, the leukocytes are removed from the plasma. As a result, the anti-microbial activity of the plasma is based only on the protein components that remain in the plasma such as complement proteins and other antimicrobial proteins. Generally, the whole blood of Anna’s Hummingbirds had more anti-microbial activity against *E*. *coli and S*. *aureus*, indicating the activity of phagocytic cells in the whole blood is important for reducing microbial growth. Because Anna’s Hummingbirds are relying on the activity of both protein and cellular components in blood, future studies should use whole blood to encompass both components.

The results of this study set the foundation for additional research to be done investigating hummingbird immunity. By understanding how age, sex and molt status affect the constitutive innate immunity of Anna’s Hummingbirds, it could be possible to predict which individuals in a population may be most vulnerable to disease. When at feeders, hummingbirds interact with an average of seven other birds. Male to male interactions tend to happen most frequently followed by male to female interactions and female to female interactions [27]. Our data indicate male Anna’s hummingbirds have higher anti-microbial activity than females. As a result of increased intraspecies interactions, male Anna’s hummingbirds may invest more in constitutive innate immunity then females. Additional research could allow us to pair the knowledge of which individuals interact the most in a feeder environment with which birds may have the lowest innate immunity at a given time in order to predict which individuals may be most likely to succumb to or spread disease in a feeder environment. The ability to make these types of predictions will be become increasingly important for conservation efforts as more species inhabit urban environments.

There is currently no published research investigating how specific diseases impact the immunity of infected hummingbirds. Avian pox is one of the most common diseases of hummingbirds in California and understanding how avian poxvirus influences the immune system of infected birds would be extremely valuable. The immune assay described in this paper requires very small amounts of blood and could be used to test the innate immune capacity of hummingbirds affected by avian pox. The avian poxvirus that infects hummingbirds is a unique subclade compared to other avian poxviruses which combined with the unique characteristics of hummingbirds make it an important disease to study [8].

The methods outlined in this study can be used to understand how additional factors may influence the constitutive innate immunity of hummingbird species. As the environments of hummingbirds become increasingly urbanized, additional threats arise that are likely to influence their health. In urbanized areas, birds are likely to be exposed to pesticides or heavy metals. In California, more than 68% of Anna’s Hummingbirds and Black-chinned Hummingbirds tested positive for at least one type of neonicotinoid insecticide [28]. Anna’s Hummingbirds in California are also known to have elevated levels of potentially harmful trace elements including iron, zinc, arsenic and mercury [29]. By sampling hummingbirds in areas where birds are known to be affected by insecticides or heavy metals, we could gain valuable information about the effects of such substances on hummingbird immunity. This is the first study to explore hummingbird immunity and serves as an important foundation for future studies examining hummingbird immunity and the specific factors that are the most influential for hummingbird health.

## Supporting information

S1 Dataset (XLSX)
